# Architecture, component, and microbiome of biofilm involved in the fouling of membrane bioreactors

**DOI:** 10.1038/s41522-016-0010-1

**Published:** 2017-02-23

**Authors:** Tomohiro Inaba, Tomoyuki Hori, Hidenobu Aizawa, Atsushi Ogata, Hiroshi Habe

**Affiliations:** Environmental Management Research Institute, National Institute of Advanced Industrial Science and Technology (AIST), 16-1 Onogawa, Tsukuba, Ibaraki 305-8569 Japan

## Abstract

Biofilm formation on the filtration membrane and the subsequent clogging of membrane pores (called biofouling) is one of the most persistent problems in membrane bioreactors for wastewater treatment and reclamation. Here, we investigated the structure and microbiome of fouling-related biofilms in the membrane bioreactor using non-destructive confocal reflection microscopy and high-throughput Illumina sequencing of 16S rRNA genes. Direct confocal reflection microscopy indicated that the thin biofilms were formed and maintained regardless of the increasing transmembrane pressure, which is a common indicator of membrane fouling, at low organic-loading rates. Their solid components were primarily extracellular polysaccharides and microbial cells. In contrast, high organic-loading rates resulted in a rapid increase in the transmembrane pressure and the development of the thick biofilms mainly composed of extracellular lipids. High-throughput sequencing revealed that the biofilm microbiomes, including major and minor microorganisms, substantially changed in response to the organic-loading rates and biofilm development. These results demonstrated for the first time that the architectures, chemical components, and microbiomes of the biofilms on fouled membranes were tightly associated with one another and differed considerably depending on the organic-loading conditions in the membrane bioreactor, emphasizing the significance of alternative indicators other than the transmembrane pressure for membrane biofouling.

## Bioreactors: Films affecting filtration

A study of factors affecting the accumulation of biofilms on filtration membranes in bioreactors should aid efforts to combat the problem. Membrane bioreactors combine filtration through a membrane with an active biological process. They are widely used in municipal and industrial wastewater treatment, but the efficiency of the filtration step is readily compromised by the growth of biofilms on the filter membranes. The nature of the microbial populations responsible for this problem, and the structure of the resulting biofilms, has not been well characterized. Tomoyuki Hori and colleagues at the Environmental Management Research Institute in Tsukuba, Japan, analyzed these issues using microscopy and sequencing of microbial DNA. They identified links between biofilm structure, chemical composition, microbial populations and the types of organic materials being treated. The increased understanding gained will guide more detailed future research.

## Introduction

Membrane bioreactors (MBRs) have been broadly exploited for the treatment of municipal and industrial wastewaters. MBRs combine the membrane separation process and activated sludge treatment, and exhibit some advantages compared with the traditional activated sludge method. Specifically, MBRs provide a smaller installation area, more efficient solid–liquid separation, less excess sludge, and higher-quality-treated wastewaters.^1, 2^ However, membrane filtration is intrinsically linked with the incidence of membrane fouling, which induces serious problems such as a decrease in the quality and quantity of the treated wastewater that results in increased operational cost.^3^

Generally, the fouling of MBRs is caused by the deposition and accumulation of inorganic and organic matters, including microbial cells (i.e., the biocake build-up), on the filtration membrane and the subsequent complete clogging of the membrane pores. Biofouling is a complex, dynamic, and relatively slow process mediated by various biological factors that are not yet thoroughly understood.^4, 5^ This situation most likely occurs as a result of the formation and development of biofilms on the filtration membrane. In recent years, microorganisms in the fouling-related biofilms have been investigated.^6–8^ However, the structural and compositional bases of the biofilms in their naturally occurring states are still unclear. To evaluate and prevent membrane biofouling, it is of particular importance to unveil the development mechanism of biofilms on filtration membranes in actual MBR runs.

Non-destructive direct observation of biofilm formation and development has recently become available due to the improvement of confocal reflection microscopy (CRM).^9, 10^ This unique analytical technique uses a special installed beam splitter to detect the light reflected from all objects, which allows three-dimensional visualization of their physical bodies. Furthermore, CRM with component-specific fluorescent probes for nucleic acids, polysaccharides, and proteins is capable of simultaneously observing the structural shapes and constituent elements, including microbial cells of biofilms. The advent of high-throughput DNA sequencers has opened a new era of microbiome studies and has generated metagenomic and gene-amplicon libraries at multimillion-sequence scales.^11, 12^ A combination of direct CRM and a comprehensive phylogenetic analysis of biofilm microbiomes should be powerful to clarify the main solid-phase components and the key microbial species involved in the fouling of MBRs, in which an enormous number of microorganism types coexist by interacting with one another.

Environmental conditions largely affect the composition and function of biofilm microbiomes.^13^ Our previous investigations showed that bacterial communities in activated sludge drastically shifted in response to organic-loading changes in an MBR,^14–16^ and their distinctive assemblages were found on fouled membranes after chemical washing.^17^ The bacterial communities on fouled membranes were herein focused due to their significant involvement in biofilm formation^13^ and were compared with the sludge bacterial communities as control. The objective in this study was to investigate the architectures, chemical components, and microbiomes of biofilms developed on filtration membranes during the actual biofouling induced at low and high organic-loading rates (OLRs) in the MBR. The examination was performed using non-destructive CRM and high-throughput Illumina sequencing of 16S rRNA genes. The relationship between the biofilm structures and microbiomes is discussed to gain deeper insights into the mechanism underlying the biofouling of the MBR.

## Results and discussion

### Physicochemical profile during the operation of a laboratory-scale MBR

To acclimatize the sludge microbiome, the MBRs were independently operated at low and high OLRs for 13 and 15 days, respectively, before collecting the fouled membrane samples (Supplementary Fig. S1). The wastewater was treated effectively under the low OLR conditions (Supplementary Fig. S1A), with the total organic carbon (TOC) of the treated effluent kept below 35 mg/L (TOC removal rates: 97.8 ± 0.4%). The transmembrane pressure (TMP) gradually increased to a maximum value of 31 kPa at the end of the operation, although the volume of the treated effluent was rather stable (ranging from 3.4 to 3.8 mL/min). Conversely, the high OLR conditions resulted in a gradual increase in the TOC of the treated effluent throughout the operation, in which the TOC removal rates decreased from 99.2 to 97.3% (Supplementary Fig. S1B). The TMP drastically increased from 3 kPa at day 3 to 34 kPa at day 5 and then increased to 44 kPa at day 10. The effluent volume decreased during the operation, with high TMPs of 30–44 kPa. After the microbiome acclimatization (Supplementary Fig. S1), under both OLR conditions, the fouled membranes were sacrificed and sampled at certain values of TMP. TMPs of 10 and 31 kPa for the low OLR conditions and those of 10, 20, 30, 44, and 50 kPa for the high OLR conditions that found in the succeeding runs were selected to examine the biofilm structures and microbiomes.

### Architecture and cell localization of biofilms on the fouled membranes

CRM was applied to non-destructively visualize the fouling-related biofilms. First, a virgin polyacrylonitrile (PAN) membrane treated with SYTO9 and propidium iodide (PI) was visualized as the control (Supplementary Fig. S2); the PAN membrane was stained with the fluorescent probes and was detectable under the microscope. Second, the live and dead cells in the biofilms were able to be distinguished by the live/dead staining in the different colors (Supplementary Fig. S3).

**Fig. 1:**
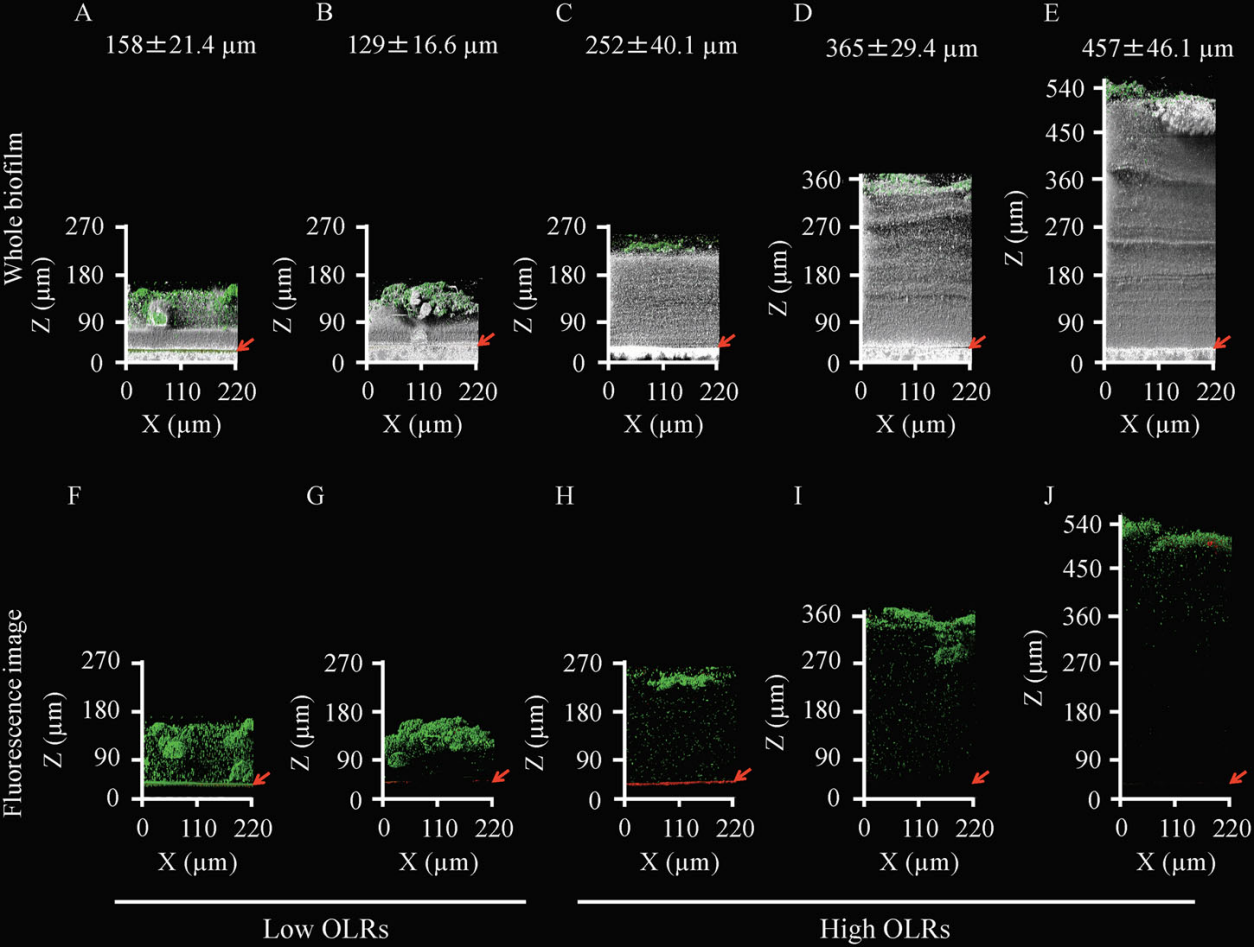
Two-dimensional architecture and thickness of fouling-related biofilms. The *gray color* indicates the physical body reflected by light, the *green* and *red colors* indicate live and dead microbial cells, respectively. Live/dead staining was performed with the SYTO9/PI dyes. The *red arrows* indicate the positions of the membrane surfaces. The average thicknesses of the biofilms are shown in the *upper part* of the figure. The *lower panels* show only fluorescent images of the biofilms. The 10 kPa (**a**, **f**) and 31 kPa (**b**, **g**) TMP biofilms under the low OLR conditions are shown on the *left*. The six panels on the *right* show biofilms at 10 kPa (**c**, **h**), 30 kPa (**d**, **i**), and 50 kPa (**e**, **j**) of TMP under the high OLR conditions. At least seven microscopic images were taken per sample, and representative images were shown for presentation. The averages of the biofilm thickness are based on at least three independent determinations, and the standard deviations are indicated

Under the low OLR conditions, the fouled membranes were obtained at two different TMPs (10 and 31 kPa) and thereafter treated with SYTO9 and PI. The direct CRM showed that the average thickness of the biofilms was approximately 150 µm at both TMPs, indicating that the biofilm thickness was not in proportion to the magnitude of the TMP (Fig. 1). The non-cell region of the biofilms indicated as a gray color in Fig. 1 seemed to be larger at 31 kPa than at 10 kPa. The fluorescence images showed that the localization of the live microbial cells was widely dispersed across the whole biofilm at the low TMP, whereas the cells were accumulated in the upper halves of the biofilm at the high TMP. The quantitative imaging analysis with the Comstat2 program indicated that 81.6 ± 5.4% of the total cells were alive even in the 31 kPa TMP biofilm. These results highlight that the change in the biofilm structures depended on the extent of membrane biofouling, even at the same OLR.

Under the high OLR conditions, the fouled membranes were obtained at five different TMPs (10, 20, 30, 44, and 50 kPa). Each fouled membrane was treated with SYTO9 and PI and then observed non-destructively (Fig. 1, Supplementary Fig. S4). In contrast to the low OLRs, the thickness of the biofilms increased linearly with the TMP values, reaching a maximum of >500 µm at the maximum pressure of 50 kPa. The biofilm thickness had a positive correlation with the TMP magnitude. Additionally, the biofilms were considerably thicker than those found at the low OLRs even when the TMPs were similar (i.e., 30 and 31 kPa) in the low and high OLRs (Fig. 1b, d). This result shows that the increase in the biofilm thickness is a significant causative agent of membrane biofouling at high OLRs. Fluorescent staining of the microbial cells and the subsequent image quantification showed that most (81.6 ± 5.4%) of the microorganisms were alive but localized specifically in the uppermost parts of the biofilms on the fouled membranes, indicating that the massive region of the thick biofilms at the high OLRs was composed of not cells but materials reflected by the light due to the direct microscopy.

### Main solid-phase components of the fouling-related biofilms

The fluorescent probes SYTO9 and PI that specifically detected DNAs inside the cell were used, but most of the biofilms were not stained, especially those found at the high OLRs. Here, staining with component-specific probes, such as the concanavalin A (ConA)-FITC conjugate, FM4-64, and FilmTracer SYPRO Ruby, was conducted to identify the main solid components of the fouling-related biofilms obtained at 31 and 50 kPa TMP under the low and high OLR conditions, respectively.

**Fig. 2:**
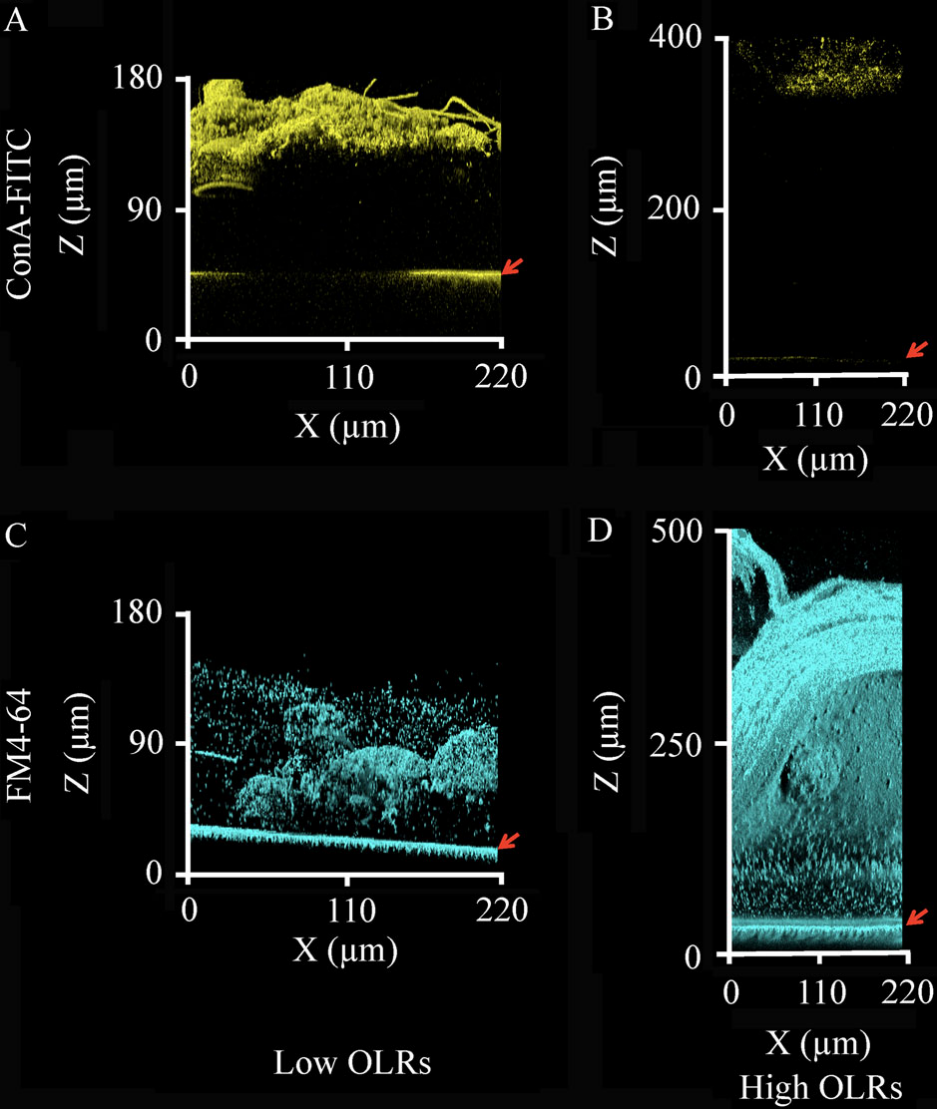
Chemical components of the fouling-related biofilms. The *yellow color* indicates polysaccharides probed by ConA-FITC, and the *cyan color* indicates the cell membrane-derived lipids stained by FM4-64. The positions of the membrane surfaces are indicated by the *red arrows. Fluorescent images* were obtained from the 31 kPa TMP biofilms under the low OLR conditions (**a**, **c**) and 50 kPa TMP biofilms under the high OLR conditions (**b**, **d**). The microscopic observations were performed on at least five different points of the fouled membranes, and the representative images are shown for presentation

Immunostaining with ConA-FITC, which specifically binds to α-Man and α-Glc of polysaccharides, showed the upper regions of the biofilms stained intensively at the low OLRs, whereas the fluorescent signal was rather weak at the high OLRs (Fig. 2a, b). This result indicated that the biofilms on fouled membranes at the low OLRs primarily stemmed from extracellular polysaccharides and live microbial cells (Figs. 1g, 2a), which was consistent with previous findings.^4, 5^ The lipophilic fluorescent probe FM4-64 was utilized to detect not only cell membrane lipids but also membrane-derived free lipids. Notably, this probe stained a large portion of the biofilms under both the low and high OLR conditions (Fig. 2c, d). The signals found under the low OLR conditions overlapped with the signals from the SYTO9-stained microbial cells (data not shown), strongly suggesting that most of the membrane lipids of living microorganisms were stained. In contrast, intensive signals were detected under the high OLR conditions despite the absence of the SYTO9 and ConA-FITC signals, demonstrating that the main solid component of the fouling-related biofilms was neither microbial cells nor extracellular polysaccharides, but free lipids. The quantitative imaging indicated that the lipids supposedly accounted for 72.3 ± 11.3% of the total biofilm amount. A large amount of the free lipids might originate from the debris of dead microorganisms because of the lack of lipids as a primary component in the synthetic wastewater supplied. Although it may be difficult to detect equally all kind of the diverse components of the biofilms and there was the possibility of the false negative results, we successfully applied these fluorescent probes to determine the main solid-phase components in the biofilms. Furthermore, staining with the fluorescent probe FilmTracer SYPRO Ruby showed a weak signal under both OLR conditions, suggesting that a slight amount of protein comprised the fouling-related biofilms (data not shown). Collectively, the non-destructive visualization underscored that the physical bodies and chemical components of the biofilms on the fouled membranes were markedly distinct depending on the OLRs used in the actual MBR runs.

### Diversity and dynamics of the biofilm microbiomes

With the microscopic visualization showing that the biofilm structures were distinctively associated with the OLRs in the MBR, high-throughput Illumina sequencing of 16S rRNA genes was utilized to investigate changes in the microbiomes on the fouled membranes. Supplementary Table S1 shows the summary of the Illumina sequence data. A total of 651,304 sequences in 27 libraries were obtained in this study, corresponding to an average of 24,112 sequences per library (minimum, 9992; maximum, 46,750). It should be noted that the live cells were mostly detected by the sequencing analysis as revealed by quantitative imaging, but the effect of the dead cells was not eliminated. A recent developed procedure^18^ to differentiate DNAs from the live and dead cells can be employed in future studies.

The α-diversity index Chao1 indicates the predicted total number of microbial species (i.e., richness), whereas the Shannon and Simpson reciprocal indices reflect the evenness and richness of microbial communities with a focus on the rare and dominant microorganisms, respectively.^19^ All of the indices at low OLRs were higher than those at high OLRs (Supplementary Table S1). Some species of the biofilm microbiomes may not have adapted to or may have died under the high OLR conditions. This possibility is supported by not only the low microbial diversity indices but also the high accumulation of free lipids that potentially resulted from dead cells on the fouled membranes (Fig. 2d). Aside from the clear difference between the low and high OLRs, the change in the α-diversity indices could be distinguished according to the TMP even within the same OLR condition. At low OLRs, the Simpson reciprocal index apparently decreased with the increase in the TMP, whereas the Chao1 and Shannon indices did not change significantly in response to the pressure (Supplementary Table S1). This result suggests that the evenness and richness of dominant microorganisms on the fouled membranes was primarily decreased, whereas those of rare microorganisms was little changed at low OLRs. At high OLRs, all of the indices exhibited increasing trends along with the increase in the TMP, indicating that the thick biofilm development might be beneficial for the diversification of both major and minor microorganisms.

**Fig. 3:**
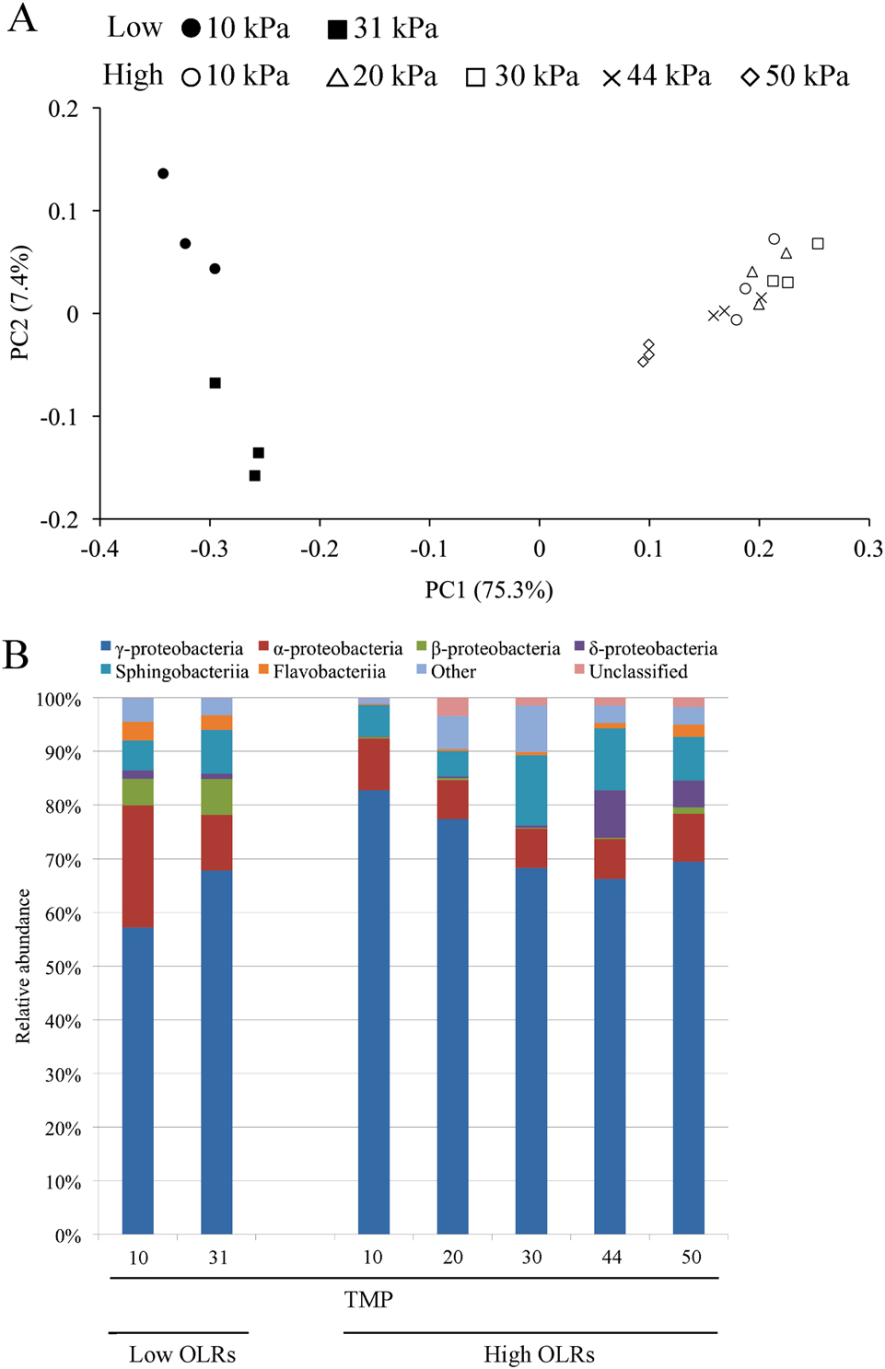
Comparison of the biofilm microbiomes at different extents of the membrane fouling. **a** Principal coordinate analysis (PCoA) scatter plot of 16S rRNA genes obtained from Illumina sequencing. The weighted UniFrac distances were calculated based on an equal number (*n* = 7114) of sequences. *Closed* and *open circles* indicate the low and high OLR conditions, respectively. The *symbol type* indicates the TMP value. **b** Class-level distribution of the fouling-related biofilm microbiomes. The relative abundances of each bacterial class are shown

Principle coordinate analysis (PCoA) of the Illumina sequence data based on weighted UniFrac distances was used to compare the whole structure of the microbiomes on the fouled membranes (Fig. 3a). The distance on the PCoA plot between the low and high OLRs was large, indicating that the biofilm microbiomes under the two tested conditions were distinct. Moreover, the microbiomes under the low OLR conditions were scattered over the left area of the plot depending on the TMP value, which might reflect the drastic decrease in the Simpson reciprocal index with the pressure increase (Supplementary Table S1). At high OLRs, the microbiomes at TMPs of 10–44 kPa were located close to the right on the plot; the microbiomes moved somewhat further to the left (Fig. 3a) when the TMP increased to 50 kPa. Because the percentage explained for PC1 was much higher than that for PC2, even the small difference in the *x*-axis position on the plot was not negligible. These results strongly suggest that the biofilm microbiomes changed along with the extent of membrane fouling under both the low and high OLR conditions.

### High-resolution phylogeny of the biofilm microbiomes

A class-level phylogenetic analysis of the Illumina sequence data was performed using the QIIME software to determine the biofilm microbiome compositions at all TMPs under the low and high OLR conditions (Fig. 3b). Class γ-Proteobacteria constituted the first majority (i.e., 57.0–82.0%) of the microbiomes for all fouled membrane samples. The other classes, such as α-, β-, and δ-Proteobacteria, Sphingobacteriia, and Flavobacteriia, exhibited subsequent dominance. Under the low OLR conditions, Flavobacteriia and β-Proteobacteria were frequently detected and accounted for 2.3–3.5 and 5.0–6.6% of the total, respectively. Under the high OLR conditions, their relative abundances were relatively small (i.e., 0.5–3.0%). In their place, class δ-Proteobacteria emerged as the major group (abundance of 8.8%) when the TMP increased to 44 kPa; the predominance of this class was maintained under the highest pressure of 50 kPa. The class-level phylogenetic characterization indicated that the biofilm microbiomes developed differently under the two tested OLR conditions in the MBR.

Because the high abundance of γ-Proteobacteria was the common characteristic under the low and high OLR conditions, the detailed composition of the biofilm microbiomes was analyzed at the species (operational taxonomic unit, OTU) level. In parallel, the sludge microbiomes were also examined as a control (Supplementary Table S2). The top 20 most abundant OTUs of the biofilm microbiomes at the highest TMPs (31 and 50 kPa) under the low and high OLR conditions, respectively, are shown in Table 1. Their increasing ratios based on the data from the 10 kPa TMP and sludge microbiomes are indicated; the former is indicative of the biofilm development and the latter is related to the growth of the microorganisms that adopted not free-living but membrane-attached lifestyles. The detailed information regarding the most highly increased OTUs and the most abundant sludge OTUs are provided as supplementary data (Supplementary Tables S2, S3, and S4).

**Table 1:** Top 20 most abundant OTUs in the developed biofilms at TMPs of 31 and 50 kPa under the low and high OLR conditions

OTU ID	Closest relative		Accession No.	Identity (%)	Relative abundance (%)	Fold change (relative to)	
	Class	Species				10 kPa	Activated sludge
Low OLR conditions							
20392	γ-proteobacteria	*Alishewanella agri*	KP282808	100	33.07	2.0**	1.1
6262	γ-proteobacteria	*Dokdonella immobilis*	NR108377	99	9.26	0.7	1.1
16852	Flavobacteriia	*Flavobacterium cucumis*	AB682419	99	4.92	2.5**	0.4**
21792	γ-proteobacteria	*Luteimonas marina*	LM643747	100	2.90	0.7**	1.0
10052	α-proteobacteria	*Brevundimonas aurantiaca*	KC855474	99	1.93	0.6	3.4
9255	γ-proteobacteria	*Alishewanella agri*	KP282808	97	1.74	1.6	1.0
22321	γ-proteobacteria	*Pseudoxanthomonas mexicana*	KM210271	99	1.36	1.0	0.6
8953	α-proteobacteria	*Gemmobacter megaterium*	NR125598	100	1.07	0.2*	0.7
20395	γ-proteobacteria	*Aquimonas voraii*	KM199274	99	1.02	0.5**	4.6
25035	γ-proteobacteria	*Pseudoxanthomonas daejeonensis*	JQ342923	97	0.97	0.3**	1.4
17206	γ-proteobacteria	*Pseudomonas mendocina*	LN835456	96	0.95	8.6	58.0
9157	Flavobacteriia	*Fluviicola hefeinensis*	JX077130	99	0.89	6.2**	29.6
24357	γ-proteobacteria	*Alishewanella agri*	KP282808	97	0.85	2.0	1.1
19777	Bacteroidia	*Marinifilum fragile*	NR044597	89	0.76	0.4**	0.5**
30089	Sphingobacteriia	*Taibaiella koreensis*	LN613111	89	0.74	0.7**	0.3*
27150	β-proteobacteria	*Albidiferax ferrireducens*	KC855480	100	0.73	4.4**	1.3
17681	α-proteobacteria	*Brevundimonas terrae*	KF818660	100	0.73	1.6	5.3
16298	γ-proteobacteria	*Alishewanella agri*	KP282808	96	0.71	1.5	1.2
19309	γ-proteobacteria	*Dokdonella immobilis*	NR108377	97	0.69	1.5	1.1
5351	δ-proteobacteria	*Klebsiella pneumoniae*	KP941758	85	0.57	0.4**	1.6**
High OLR conditions							
17206	γ-proteobacteria	*Pseudomonas mendocina*	LN835456	96	42.15	0.6**	0.8*
25035	γ-proteobacteria	*Pseudoxanthomonas daejeonensis*	JQ342923	97	4.81	5.9**	3.1**
8864	Sphingobacteriia	*Lewinella nigricans*	NR115013	89	4.62	1.2	0.8
30480	δ-proteobacteria	*Pelobacter acidigallici*	NR026154	86	4.22	43.8**	38.7**
20392	γ-proteobacteria	*Alishewanella agri*	KP282808	100	4.11	9.7**	4.9**
1879	Sphingobacteriia	*Parapedobacter koreensis*	NR043933	96	2.73	1.5**	0.3**
17375	γ-proteobacteria	*Pseudomonas tuomuerensis*	KP973997	96	2.17	1.0	0.9
28892	Actinobacteria	*Naumannella halotolerans*	KP798854	82	1.81	21.8*	17.1*
25515	Flavobacteriia	*Imtechella halotolerans*	NR117181	94	1.75	17.9**	4.8**
6262	γ-proteobacteria	*Dokdonella immobilis*	NR108377	99	1.68	10.3**	6.6**
13461	α-proteobacteria	*Plastorhodobacter daqingensis*	KF312715	99	1.67	0.3**	0.2*
15507	γ-proteobacteria	*Stenotrophomonas acidaminiphila*	LN848748	100	0.86	2.6**	2.3**
15641	δ-proteobacteria	*Bdellovibrio exovorus*	NR102876	100	0.81	8.7^a^	494.3
27770	α-proteobacteria	*Pelagibacterium halotolerans*	KJ939460	99	0.77	2.2**	0.9
29369	γ-proteobacteria	*Pseudomonas mendocina*	LN835456	94	0.67	1.5	1.6
8953	α-proteobacteria	*Gemmobacter megaterium*	NR125598	100	0.61	5.0**	5.4**
20395	γ-proteobacteria	*Aquimonas voraii*	KM199274	99	0.55	13.9**	3.2**
10305	γ-proteobacteria	*Gemmobacter aquatilis*	KM220014	100	0.52	2.3**	2.1**
29195	α-proteobacteria	*Paracoccus pantotrophus*	KT715779	97	0.52	2.9**	1.4
20393	α-proteobacteria	*Falsirhodobacter deserti*	KF268394	98	0.51	0.9	0.4

^*^ *P* < 0.1, ^**^ *P* < 0.05

^a^No sequence was detected in the 10 kPa TMP biofilm microbiomes, and the value indicates fold changes related to the 30 kPa TMP biofilm microbiomes

In the 31 kPa TMP biofilm microbiomes under the low OLR conditions, the most abundant OTU 20392 (*Alishewanella agri* [accession no. KP282808; 100% sequence similarity]) was also the highest-ranking OTU among the sludge microbiomes (Table 1 and Supplementary Table S2), which possibly affected its predominance on the fouled membranes. However, another three *Alishewanella* OTUs (9255, 24357, and 16298) dominated the biofilm microbiomes and a variety of *Alishewanella* species were detected with great frequency among the most highly increased OTUs (Supplementary Tables S3 and S4). *Alishewanella* species are known to be biofilm formers.^20^ These results imply that the genus *Alishewanella* proliferated drastically on the fouled membranes and played a pivotal role in the development of the polysaccharide-rich biofilms (Fig. 2a). Apart from *Alishewanella* spp., some of the dominant microorganisms (i.e., OTUs 10052, 20395, 17206, 9157, and 17681) exhibited higher increasing ratios based on the sludge microbiomes than those based on the 10 kPa TMP microbiomes, indicating that these OTUs were most likely responsible for the formation rather than the development of the biofilms on the fouled membranes. For details, the closest relative of OTU 9157 (*Fluviicola hefeinensis* [JX077130; 99% similarity]) belonging to class Flavobacteriia was detected in the fouling processes of a forward osmosis membrane^7^ and co-existed in reed periphyton (biofilm) communities with *Aquimonas voraii* (KM199274; a relative of the dominant OTU 20395) and *Rhenheimera chironomi* (LC054836; a relative of the highly increased OTU 26015 in Supplementary Table S4).^21^ Furthermore, the OTUs 10052 and 17681 were affiliated within the genus *Brevundimonas*, which is widely recognized as the primary microorganisms of fouled MBR membranes.^22^ Among them, *B. aurantiaca* (the closest relative of OTU 10052) has been reported to produce extracellular polysaccharides and proteins, which resulted in the fouling of membrane microfilters.^23^ In contrast, the β-proteobacterial OTU 27150 (*Albidiferax ferrireducens* [KC855480; 100% similarity]), which is a biofilm former in microbial fuel cells,^24^ showed high increasing ratios, especially based on the 10 kPa TMP microbiomes, indicating its relatively high contribution to biofilm development.

Conversely, in the 50 kPa TMP biofilm microbiomes under the high OLR conditions, the most abundant microorganism was OTU 17206 (*Pseudomonas mendocina* [LN835456; 96% similarity]), which accounted for more than 40% of the total (Table 1). The other pseudomonads were also dominant on the fouled membranes and were frequently detected among the most highly increased OTUs (Supplementary Tables S3 and S4). It has been so far reported that the pseudomonads abundantly existed in the biofilm on filtration membrane in MBRs.^7^ *P. mendocina* has been reported to form the biofilm architecture,^25^ and the pseudomonads, especially *P. aeruginosa*, are well known as model pure-culture organisms in biofilm studies.^26^ Although highly abundant, OTU 17206 decreased with the increase in the TMP. For details, the quantitative imaging analysis revealed that the amounts of the total cells were determined to be 3.72 ± 1.39 and 2.18 ± 0.82 µm^3^/µm^2^ in the 10 and 50 kPa TMP biofilms, respectively. Based on the total cell amounts and the relative abundance of OTU 17206, it was calculated that the absolute quantity of this OTU was significantly low in the 50 kPa biofilm (0.92 µm^3^/µm^2^) compared with that in the 10 kPa biofilm (2.60 µm^3^/µm^2^). The lowered quantities of the other OTUs were not apparent. The results indicated that the free lipids on fouled membranes (Fig. 2d) most likely originated from the debris of the dominant pseudomonads OTU 17206, thus it was essential for biofilm development on fouled membranes under the high OLR conditions. Apart from these pseudomonads, the δ-proteobacterial OTU 30480 (*Pelobacter acidigallici* [NR026154; 86%]), the actinobacterial OTU 28892 (*Naumannella halotolerans* [KP798854; 82%]), and the γ-proteobacterial OTU 22289 (*Alkanindiges hongkongensis* [NR115179; 87%]) showed dominance on fouled membranes and/or high increasing ratios (Table 1 and Supplementary Tables S3 and S4). The relatives of these OTUs are potential biofilm formers;^22, 27–29^ however, all of the sequence similarities were quite low (i.e., 82–87%), which made it difficult to estimate their true ecophysiological roles. Nevertheless, the phylogenetically novel bacteria might be tightly associated with biofilm formation and development due to their dominance and proliferation on fouled membranes. The OTUs 25515 and 6262, which had high increasing rates based on both the initial biofilm and sludge microbiomes, were related to *Imtechella halotolerans* (NR117181; 94% similarity) and *Dokdonella immobilis* (NR108377; 99% similarity), respectively. These relative species have been reported to possess lipase activities,^30, 31^ suggesting that these dominant OTUs were stimulated by the free lipids that accumulated in the fouling-related biofilms (Fig. 2d). Finally, it is also vital to point out that the δ-proteobacterial OTU 27770 (*Bdellovibrio exovorus* [NR102876; 100% similarity]) exhibited the highest increasing ratio (i.e., 494.3 fold) based on the data from the sludge microbiomes, although the bacteria were not detectable (i.e., almost entirely absent with a relative abundance <0.005%) in the 10 and 20 kPa TMP biofilm microbiomes. *B. exovorus* was identified as an obligate predator with the ability to obtain nutrients from the cytosol of other bacteria but not their cell membranes.^32^ The physiological character of *B. exovorus* apparently contributed to the accumulation of the free lipids (Fig. 2d) that were likely derived from the dead cells of OTU 17206 in the biofilms. Because the fouling-related biofilms were clearly in a static state (Figs. 1, 2) compared with the dynamic liquid phases with aeration in the MBR, this situation seems to be advantageous for the ability of *B. exovorus* to prey and proliferate. *Bdellovibrio* spp. was recently reported to directly attack and reduce the biofilms formed by *P. aeruginosa* (a relative of the most abundant OTU 17206)^33^ and *Stenotrophomonas maltophilia* (a relative of the dominant OTUs 15507 and 25035).^34–36^ In this regard, *Bdellovibrio*-related species also occurred simultaneously with the relatives of the OTUs 6262, 15507, and 25035 in a packed bed biofilm reactor.^37^ Other potential predator bacteria (i.e., OTUs 5506 [*Acholeplasma laidlawii* (KP742977; 100%)], 29658 and 20130 [*Leadbetterrella byssophila* (NR074303; 94%)]) were frequently detected as highly increased species relative to the sludge microbiomes (Supplementary Table S4).^38–40^ These results indicate that the bacterial predator–prey interaction may be a fundamental factor that contributes to the formation and development of the thick biofilm microbiomes in MBRs. Moreover, clarifying the effects of the food web across the phylogenetic kingdoms, i.e., Bacteria and Eukarya, on the biofilm formation and development warrants further investigation.

## Conclusion

Due to the considerable difficulty in the comprehensive and rigorous analysis of biofilms in natural and engineered environments, biofilm structures and microbiomes in their naturally occurring states remain largely unknown. To explore the developmental mechanism of biofilms on filtration membranes that may contribute to the resolution of the most persistent biofouling problem of wastewater treatment and reclamation, direct CRM was implemented to non-destructively visualize the fouling-related biofilms, followed by high-resolution phylogenetic identification of the biofilm microbiomes using high-throughput Illumina sequencing. By employing the combined approach, we demonstrated for the first time the clear divergence in the three-dimensional architectures, chemical components, and microbiomes of fouling-related biofilms produced under two distinct organic-loading conditions during actual MBR runs. The results of this study clearly revealed that the formation and development of fouling-related biofilms in MBRs were linked to various environmental factors, including the deposition and accumulation of chemical compounds (e.g., extracellular polysaccharides and membrane-derived free lipids) and the species composition and interspecies relationships of the biofilm microbiomes. To elucidate the detailed mechanisms underlying biofouling in MBRs, the ecophysiology and functional gene expression of the biofilm microbiomes specifically localized to the fouled membranes should be investigated.

## Materials and methods

### Experimental setup and operation of a laboratory-scale MBR

The laboratory-scale MBR used in this study consisted of three compartments and had a total volume of 28.0 L. The schematic view of the reactor was reported previously.^15^ Air was supplemented at a flow rate of 10 L/min through a diffuser set in each compartment to maintain dissolved oxygen (DO) values from 1.0 to 8.0 mg/L and to continuously agitate the activated sludge. A flat membrane module (M-fine; Awa Paper Mfg, Co., Tokushima, Japan) made of PAN was submerged in the third compartment during the operation. The effective area of the filtration membrane was 0.012 m^2^ with a pore size of 0.07 μm. The membrane module was operated with a permeate extraction cycle for 9 min and a pause for 1 min to improve the membrane performance by intermittent suction. In previous study,^41^ this method was proven effective in preventing membrane fouling due to the enhanced foulant back transport under pressure relaxation. The membrane surface was continuously washed with air bubbles to reduce membrane fouling. The synthetic wastewater stored at 4 °C was supplied into the reactor at a flow rate of 5.3 L/day. The effluent-flowing rate for the membrane-filtrated water was adjusted to 5.3 L/day, resulting in a hydraulic retention time of 6 days. The reactor was started up with the inoculation of an activated sludge obtained from a municipal wastewater treatment plant (Kinu aqua-station, Ibaraki, Japan). The return flow rate of the sludge from the third to the first compartment in the MBR was 28.8 L/day. Two different OLRs of 46.5 and 93.0 mg TOC/L/day were conducted from the beginning of the operation. The composition of the synthetic wastewater used for the low OLRs was as follows: CH_3_COONa (2.65 g/L), NH_4_Cl (0.376 g/L), KH_2_PO_4_ (0.109 g/L), peptone (0.706 g/L), FeCl_3_•6H_2_O (0.782 mg/L), CaCl_2_ (1.56 mg/L), MgSO_4_ (1.56 mg/L), KCl (1.56 mg/L), and NaCl (1.56 mg/L). For the high OLRs, the synthetic wastewater components were twice the concentrations of those used to generate the low OLR wastewater. The TOC values of 1130 and 2260 mg/L corresponded to 450 and 900 mg/L, respectively, of the chemical oxygen demand (COD). Before experiment, it has been confirmed that microbiome composition was stabilized for approximately 10 days^14–16^ and membrane fouling occurred under both the low and high OLR conditions. The MBRs were independently run under both the OLR conditions and were operated for enough times (i.e., 13 and 15 days at low and high OLRs) before collecting the fouled membrane samples.

### Chemical analysis procedures

The mixed liquor suspended solid, temperature, DO, and pH in all three compartments of the MBR as well as the TMP of the membrane module were monitored throughout the operation. The liquid and solid phases of the activated sludges sampled from the first and second compartments were separated by centrifugation (15,300×*g*, 15 min, 4 °C) and the supernatants were filtered using a cellulose acetate membrane (ø0.20 μm, ADVANTEC, Tokyo, Japan). The TOC and total nitrogen (TN) concentrations in the supernatants and treated effluent were analyzed using a TOC-TN analyzer (TOC-L/TNM-L; Shimadzu, Kyoto, Japan). The COD value was measured with a COD analyzer (DR2800 and DRB200; Hach, CO, USA) and an appropriate kit (TNT820 or TNT821; Hach).

### Biofilm visualization and quantitative imaging analysis

After picking up the membrane module from MBR, whole fouled membranes were dismounted and thereafter virgin membranes were installed for next sampling. The sampled fouled membranes were temporarily stored at 4 °C and were promptly treated for microscopic visualization. The direct CRM performed for biofilm visualization was described previously.^9, 10^ Briefly, a laser scanning microscope (LSM880; Carl Zeiss, Jena, Germany) equipped with a 40x/0.80 numerical aperture Achroplan W water immersion objective (Carl Zeiss) was used to acquire the biofilm images. Fouled membrane samples were treated for 15 min with SYTO9 (Molecular Probes, OR, USA) and PI (Sigma-Aldrich, MO, USA) at final concentrations of 5 and 15 µM, respectively. SYTO9 and PI stained the live and dead microbial cells. The other staining dyes (i.e., ConA-FITC conjugate [Sigma-Aldrich], FM4-64 [Molecular Probes], and the FilmTracer SYPRO Ruby biofilm matrix stain [Molecular probes]) were used to visualize polysaccharides, lipids (derived from the plasma membrane), and proteins, respectively, according to the manufacturers’ instructions. Owing to broad and successful application of labeling with these probes to biofilm researches,^42, 43^ the stringent conditions for CRM were carefully established and the microscopic observation was precisely done. The fluorescent probes-treated membranes were washed with water once, and set in the sample chamber filled with water, which can eliminate the non-specific binding dye and residual dye that can cause false positive results. Then, the membranes were observed under a water immersion lens without a cover glass for their non-destructive visualization. By immersing the membranes in water, distortions of the thick biofilm image are suppressed to negligible levels. Illumination with 488 and 514 nm argon lasers was conducted to detect the SYTO9/FITC and FM4-64 fluorescence, respectively. The PI fluorescence was detected with a 543 nm He-Ne laser. Reflected lights were obtained using a 488-nm argon laser. The autofluorescnece was not detected from PAN membrane, and there was a little FITC-like autofluorescence in the biofilms. An MBS T80/R20 filter (Carl Zeiss) was used as the main beam splitter to detect the reflected light. The obtained confocal images were analyzed using the ZEN software (Carl Zeiss). At least seven microscopic images were taken per sample and representative images were shown. The average biofilm thicknesses are based on three or more independent determinations, and the standard deviations are indicated. The live and dead cell amounts were calculated from the fluorescent signal intensities of SYTO9 and PI, respectively, by using Comstat2 program (www.comstat.dk).^44, 45^ In addition, the ratios of the free lipids to the total biofilm amounts were estimated based on the comparison between the FM4-64 fluorescent signal and reflected signal intensities. Because the reflected signal is known to be affected by noise, more rigorous optimization of reflection microscopy is needed for more exact quantitative imaging of the biofilms in future. The determination was done on the basis of three to ten independent images analysis, and the standard deviation was obtained.

### DNA extraction and PCR amplification

One hundred mm^2^ pieces of the fouled membranes and activated sludge were sampled and stored at −20 °C. Total DNA was extracted from the stored membrane pieces and activated sludges, according to a direct lysis protocol that included bead-beating.^46^

Three replicates were conducted for the DNA extraction and the subsequent protocols to analyze each fouled membrane and activated sludge sample. RNA was digested with Type II-A ribonuclease (Sigma-Aldrich). The purified DNA was quantified using a NanoDrop Lite (Thermo Fisher Scientific, MA, USA) and was used as the template for PCR amplification with a high fidelity DNA polymerase (Q5; NEB, MA, USA). The V4 region of 16S rRNA genes was amplified with the 515F and 806R primers^12^ modified for multiplex sequencing as reported previously.^11^ The PCR conditions were as follows: initial denaturation at 98 °C for 90 s, 35–40 cycles of denaturation at 98 °C for 10 s, annealing at 56–58 °C for 30 s and extension at 72 °C for 30 s, and a final extension step at 72 °C for 2 min.

### High-throughput Illumina sequencing of the 16S rRNA gene amplicons

High-throughput Illumina sequencing was performed as described previously.^47^ Briefly, the PCR product was first purified with an AMPure XP kit (Beckman Coulter, CA, USA) and then with a QIAquick gel extraction kit (Qiagen, Venlo, The Netherlands). The DNA concentration was determined spectrophotometrically with a Quant-iT PicoGreen dsDNA reagent (Life Technologies, CA, USA) and a NanoDrop 3300 (Thermo Fisher Scientific). An appropriate amount of the 16S rRNA gene amplicon and an internal control (PhiX Control V3; Illumina, CA, USA) were subjected to paired-end sequencing with a 300-cycle MiSeq reagent kit (Illumina) and a MiSeq sequencer (Illumina). The removal of the PhiX, low-quality (Q < 30) and chimeric sequences and the assembly of the paired-end reads were performed as described in the previous study.^48^ The sequences in each library were phylogenetically characterized using the QIIME software.^49^ Alpha-diversity indices (i.e., Chao1, Shannon, and Simpson reciprocal) and the weighted UniFrac distances for the PCoA were calculated based on an equal number (*n* = 7114) of sequences using the QIIME software. The closest relative of the OTU with a cutoff value of 97% sequence similarity was determined by an NCBI BLAST search (http://blast.ncbi.nlm.nih.gov/). The raw sequence data in this study were deposited in the sequence read archive in the DDBJ database (http://www.ddbj.nig.ac.jp/) under the accession numbers DRA004744.

## Electronic supplementary material

Supplementary Information

Supplementary Figure S1

Supplementary Figure S2

Supplementary Figure S3

Supplementary Figure S4

Supplementary Table S1

Supplementary Table S2

Supplementary Table S3

Supplementary Table S4

## References

[1] Judd S (2008). The status of membrane bioreactor technology. Trends Biotechnol..

[2] Visvanathan C, Ben Aim R, Parameshwaran K (2000). Membrane separation bioreactors for wastewater treatment. Crit. Rev. Env. Sci. Technol..

[3] Wang Z, Wu Z, Tang S (2009). Extracellular polymeric substances (EPS) properties and their effects on membrane fouling in a submerged membrane bioreactor. Water Res..

[4] Baker JS, Dudley LY (1998). Biofouling in membrane systems—a review. Desalination.

[5] Drews A, Lee C-H, Kraume M (2006). Membrane fouling—a review on the role of EPS. Desalination.

[6] Vanysacker L, Boerjan B, Declerck P, Vankelecom IFJ (2014). Biofouling ecology as a means to better understand membrane biofouling. Appl. Microbiol. Biotechnol..

[7] Zhang Q (2014). Characterization of biofouling in a lab-scale forward osmosis membrane bioreactor (FOMBR). Water Res..

[8] Ziegler AS (2016). Dynamics of the fouling layer microbial community in a membrane bioreactor. PLoS ONE.

[9] Inaba T (2013). Three-dimensional visualization of mixed species biofilm formation together with its substratum. Microbiol. Immunol..

[10] Yawata Y (2010). Monitoring biofilm development in a microfluidic device using modified confocal reflection microscopy. J. Biosci. Bioeng..

[11] Bartram AK, Lynch MDJ, Stearns JC, Moreno-Hagelsieb G, Neufeld JD (2011). Generation of multimillion-sequence 16S rRNA gene libraries from complex microbial communities by assembling paired-end illumina reads. Appl. Environ. Microbiol..

[12] Caporaso JG (2012). Ultra-high-throughput microbial community analysis on the Illumina HiSeq and MiSeq platforms. ISME J..

[13] Hall-Stoodley L, Costerton JW, Stoodley P (2004). Bacterial biofilms: from the natural environment to infectious diseases. Nat. Rev. Microbiol..

[14] Sato Y, Hori T, Navarro RR, Habe H, Ogata A (2016). Functional maintenance and structural flexibility of microbial communities perturbed by simulated intense rainfall in a pilot-scale membrane bioreactor. Appl. Microbiol. Biotechnol..

[15] Sato Y (2016). Fine-scale monitoring of shifts in microbial community composition after high organic loading in a pilot-scale membrane bioreactor. J. Biosci. Bioeng..

[16] Sato, Y. et al. Effects of organic-loading-rate reduction on sludge biomass and microbial community in a deteriorated pilot-scale membrane bioreactor. *Microbes Environ.* **31**, 361–364 (2016). 10.1264/jsme2.ME16015 PMC5017815 27431196

[17] Navarro RR (2016). High-resolution phylogenetic analysis of residual bacterial species of fouled membranes after NaOCl cleaning. Water Res..

[18] Chiao T-H, Clancy TM, Pinto A, Xi C, Raskin L (2014). Differential resistance of drinking water bacterial populations to monochloramine disinfection. Environ. Sci. Technol..

[19] Hill MO (1973). Diversity and evenness: a unifying notation and its consequences. Ecology.

[20] Kim J, Jung J, Sung J-S, Chun J, Park W (2012). Genome sequence of pectin-degrading alishewanella agri, isolated from landfill soil. J. Bacteriol..

[21] Rusznyak A, Vladar P, Szabo G, Marialigeti K, Borsodi AK (2008). Phylogenetic and metabolic bacterial diversity of *Phragmites australis* periphyton communities in two Hungarian soda ponds. Extremophiles.

[22] Zheng X, Ernst M, Huck PM, Jekel M (2010). Biopolymer fouling in dead-end ultrafiltration of treated domestic wastewater. Water Res..

[23] Badireddy AR, Chellam S, Yanina S, Gassman P, Rosso KM (2008). Bismuth dimercaptopropanol (BisBAL) inhibits the expression of extracellular polysaccharides and proteins by *Brevundimonas diminuta*: implications for membrane microfiltration. Biotechnol. Bioeng..

[24] Liu ZD (2007). Improving energy accumulation of microbial fuel cells by metabolism regulation using *Rhodoferax ferrireducens* as biocatalyst. Lett. Appl. Microbiol..

[25] Mangwani N, Shukla SK, Rao TS, Das S (2014). Calcium-mediated modulation of *Pseudomonas mendocina* NR802 biofilm influences the phenanthrene degradation. Colloids Surf. B.

[26] O’Toole GA, Kolter R (1998). Flagellar and twitching motility are necessary for *Pseudomonas aeruginosa* biofilm development. Mol. Microbiol..

[27] Macedo AJ, Timmis KN, Abraham W-R (2007). Widespread capacity to metabolize polychlorinated biphenyls by diverse microbial communities in soils with no significant exposure to PCB contamination. Environ. Microbiol..

[28] Rieser G, Scherer S, Wenning M (2012). *Naumannella halotolerans* gen. nov., sp. nov., a gram-positive coccus of the family Propionibacteriaceae isolated from a pharmaceutical clean room and from food. Int. J. Syst. Evol. Microbiol..

[29] Szewzyk U, Schink B (1988). Surface colonization by and life-cycle of pelobacter-acidigallici studied in a continuous-flow microchamber. J. Gen. Microbiol..

[30] Liu Y, Jin JH, Liu HC, Liu ZP (2013). *Dokdonella immobilis* sp. nov., isolated from a batch reactor for the treatment of triphenylmethane dye effluent. Int. J. Syst. Evol. Microbiol..

[31] Surendra V, Bhawana P, Suresh K, Srinivas TN, Kumar PA (2012). *Imtechella halotolerans* gen. nov., sp. nov., a member of the family Flavobacteriaceae isolated from estuarine water. Int. J. Syst. Evol. Microbiol..

[32] Koval SF (2013). *Bdellovibrio exovorus* sp nov., a novel predator of Caulobacter crescentus. Int. J. Syst. Evol. Microbiol..

[33] Iebba, V. et al. Bdellovibrio bacteriovorus directly attacks *Pseudomonas aeruginosa* and *Staphylococcus aureus* cystic fibrosis isolates. *Front. Microbiol.* **5**, 1–9 (2014). 10.3389/fmicb.2014.00280 PMC4046265 24926292

[34] Chanyi, R. M., Koval, S. F. & Brooke, J. S. *Stenotrophomonas maltophilia* biofilm reduction by Bdellovibrio exovorus. *Environ. Microbiol. Rep.* **8**, 343–351(2016). 10.1111/1758-2229.12384 26929093

[35] Mangwani N, Shukla SK, Kumari S, Rao TS, Das S (2014). Characterization of *Stenotrophomonas acidaminiphila* NCW-702 biofilm for implication in the degradation of polycyclic aromatic hydrocarbons. J. Appl. Microbiol..

[36] Trujillo-Cabrera Y, Ponce-Mendoza A, Vásquez-Murrieta MS, Rivera-Orduña FN, Wang ET (2012). Diverse cellulolytic bacteria isolated from the high humus, alkaline-saline chinampa soils. Ann. Microbiol..

[37] Chen C-Y, Yen S-H, Chung Y-C (2014). Combination of photoreactor and packed bed bioreactor for the removal of ethyl violet from wastewater. Chemosphere.

[38] Abt B (2011). Complete genome sequence of Leadbetterella byssophila type strain (4M15). Stand. Genomic Sci..

[39] Hanajima D, Aoyagi T, Hori T (2015). Survival of free-living Acholeplasma in aerated pig manure slurry revealed by (13)C-labeled bacterial biomass probing. Front. Microbiol..

[40] Pasternak Z (2013). By their genes ye shall know them: genomic signatures of predatory bacteria. ISME J..

[41] Hong SP, Bae TH, Tak TM, Hong S, Randall A (2002). Fouling control in activated sludge submerged hollow fiber membrane bioreactors. Desalination.

[42] Strathmann M, Wingender J, Flemming H-C (2002). Application of fluorescently labelled lectins for the visualization and biochemical characterization of polysaccharides in biofilms of *Pseudomonas aeruginosa*. J. Microbiol. Methods.

[43] Ma L (2009). Assembly and development of the *Pseudomonas aeruginosa* biofilm matrix. PLoS Pathog..

[44] Heydorn A (2000). Quantification of biofilm structures by the novel computer program COMSTAT. Microbiology.

[45] Vorregaard, M. *Comstat2—A Modern 3D Image Analysis Environment for Biofilms* (Technical University of Denmark, 2008).

[46] Noll M, Matthies D, Frenzel P, Derakshani M, Liesack W (2005). Succession of bacterial community structure and diversity in a paddy soil oxygen gradient. Environ. Microbiol..

[47] Aoyagi T (2015). Ultra-high-sensitivity stable-isotope probing of rRNA by high-throughput sequencing of isopycnic centrifugation gradients. Environ. Microbiol. Rep..

[48] Itoh, H. *et al.*. Bacterial population succession and adaptation affected by insecticide application and soil spraying history. *Front. Microbiol.* **5**, 1–12 (2014). 10.3389/fmicb.2014.00457 PMC4148734 25221549

[49] Caporaso JG (2010). QIIME allows analysis of high-throughput community sequencing data. Nature Methods.

