# Supplementary material for: Architecture, component, and microbiome of biofilm involved in the fouling of membrane bioreactors
Source: NPJ Biofilms Microbiomes. 2017 Feb 23;3:5. doi: 10.1038/s41522-016-0010-1 (PMC5445582; doi:10.1038/s41522-016-0010-1)
Supplement: Supplementary file 1 — Supplementary Information [file 41522_2016_10_MOESM1_ESM.docx]

**Supplemental Table S1.** Summary of the Illumina sequencing data

^a^Calculated from the equation C_X_ = 1 - (n/N), where “n” is the number of OTUs composed of singletons, and N is the total number of sequences.

^b^Each index was calculated based on an equal number of sequences (n = 7114). All data were based on the average of 3 replicates.

**Supplemental Table S2.** Top 20 most abundant OTUs in the activated sludge microbiome under the low and high OLR conditions

**Supplemental Table S3.** Top 20 most highly increased OTUs in the developed biofilm microbiomes at TMPs of 31 and 50 kPa relative to the 10 kPa TMP biofilm microbiomes under the low and high OLR conditions

*P<0.1, **P<0.05

**Supplemental Table S4.** Top 20 most highly increased OTUs in the developed biofilm microbiomes at TMPs of 31 and 50 kPa relative to the activated sludge microbiomes under the low and high OLR conditions

*P<0.1, **P<0.05

**Supplemental Figure S1.** Change in physicochemical parameters during the microbiome-acclimatizing operation in MBRs. The open circle, open triangle and closed circle indicate the TMP, TOC and effluent rate, respectively. (A) Low OLR conditions and (B) High OLR conditions.

**Supplemental Figure S2.** Staining of a virgin filtration membrane as a control. The grey color indicates the reflected light image (A). The green and red colors indicate regions stained by SYTO9 (B) and PI (C), respectively.

**Supplemental Figure S3.** Cross-section images of biofilms stained by SYTO9 and PI. (A) 31 kPa TMP biofilm at low OLRs and (B) 50 kPa TMP biofilm at high OLRs. The green and red colors indicate the live and dead microbial cells, respectively. The bars indicate 30 µm. These images were shown as the representative in the image stacks of the biofilms.

**Supplemental Figure S4.** Three-dimensional images of the fouling-related biofilms. (A) 31 kPa TMP biofilm at low OLRs and (B) 50 kPa TMP biofilm at high OLRs. The grey color indicates the physical body reflected by light. The green and red colors indicate the live and dead microbial cells, respectively. These images were another angle of the same images which were shown in Figure 1B and 1E.
