# Supplementary figures and images for: Architecture, component, and microbiome of biofilm involved in the fouling of membrane bioreactors
Source: NPJ Biofilms Microbiomes. 2017 Feb 23;3:5. doi: 10.1038/s41522-016-0010-1 (PMC5445582; doi:10.1038/s41522-016-0010-1)

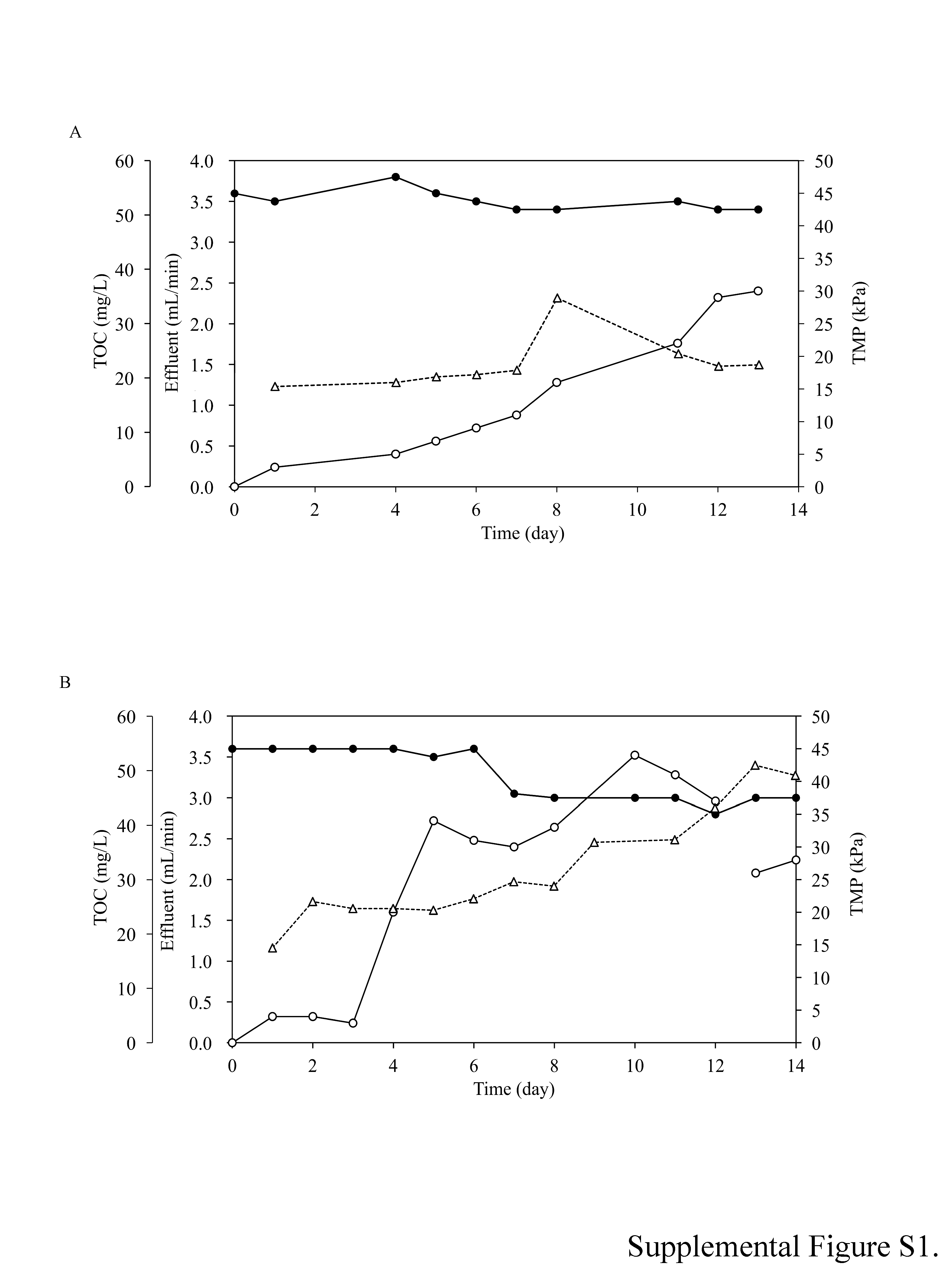

Supplement: Supplementary file 2 — Supplementary Figure S1 [file 41522_2016_10_MOESM2_ESM.tif]

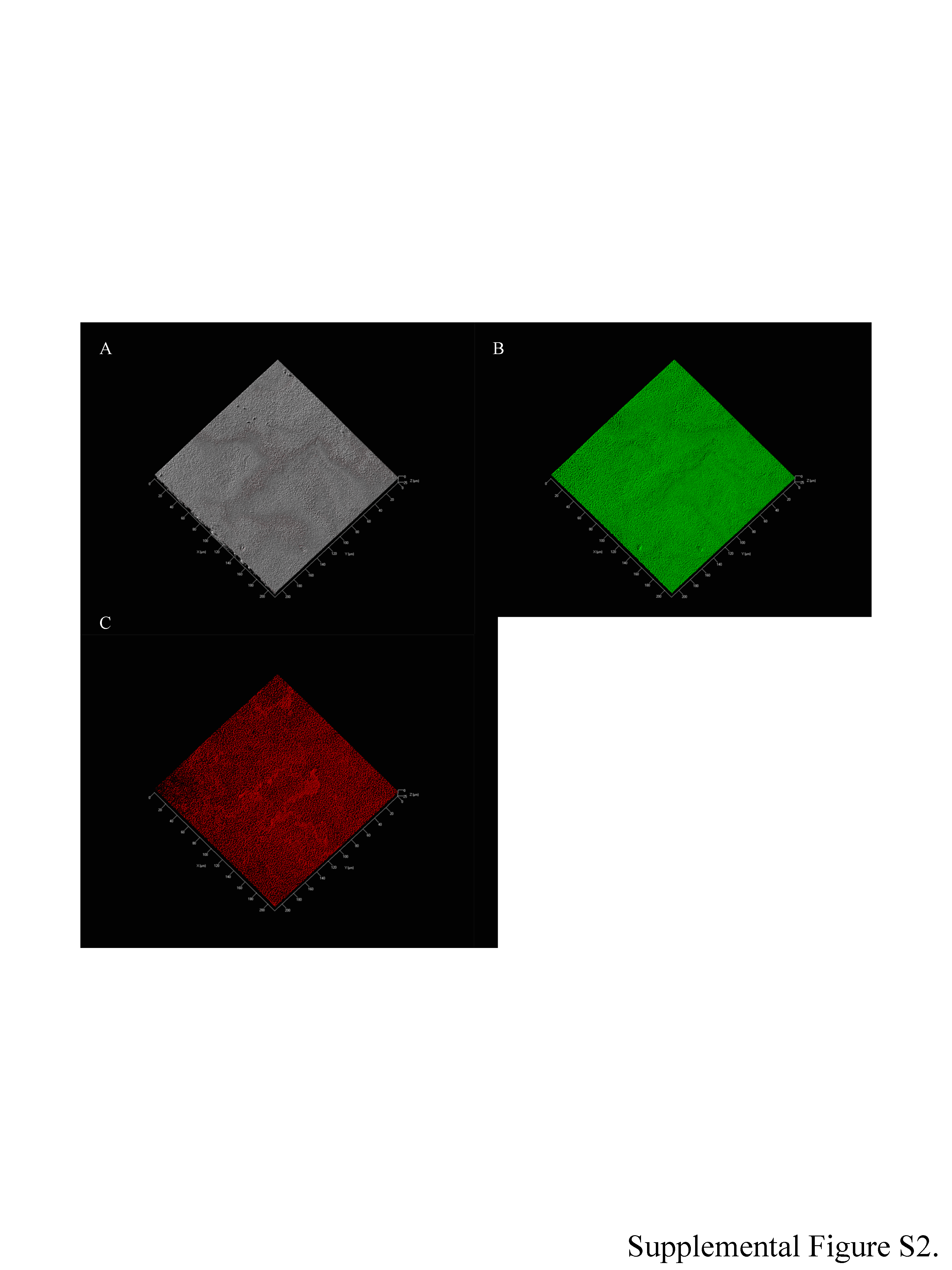

Supplement: Supplementary file 3 — Supplementary Figure S2 [file 41522_2016_10_MOESM3_ESM.tif]

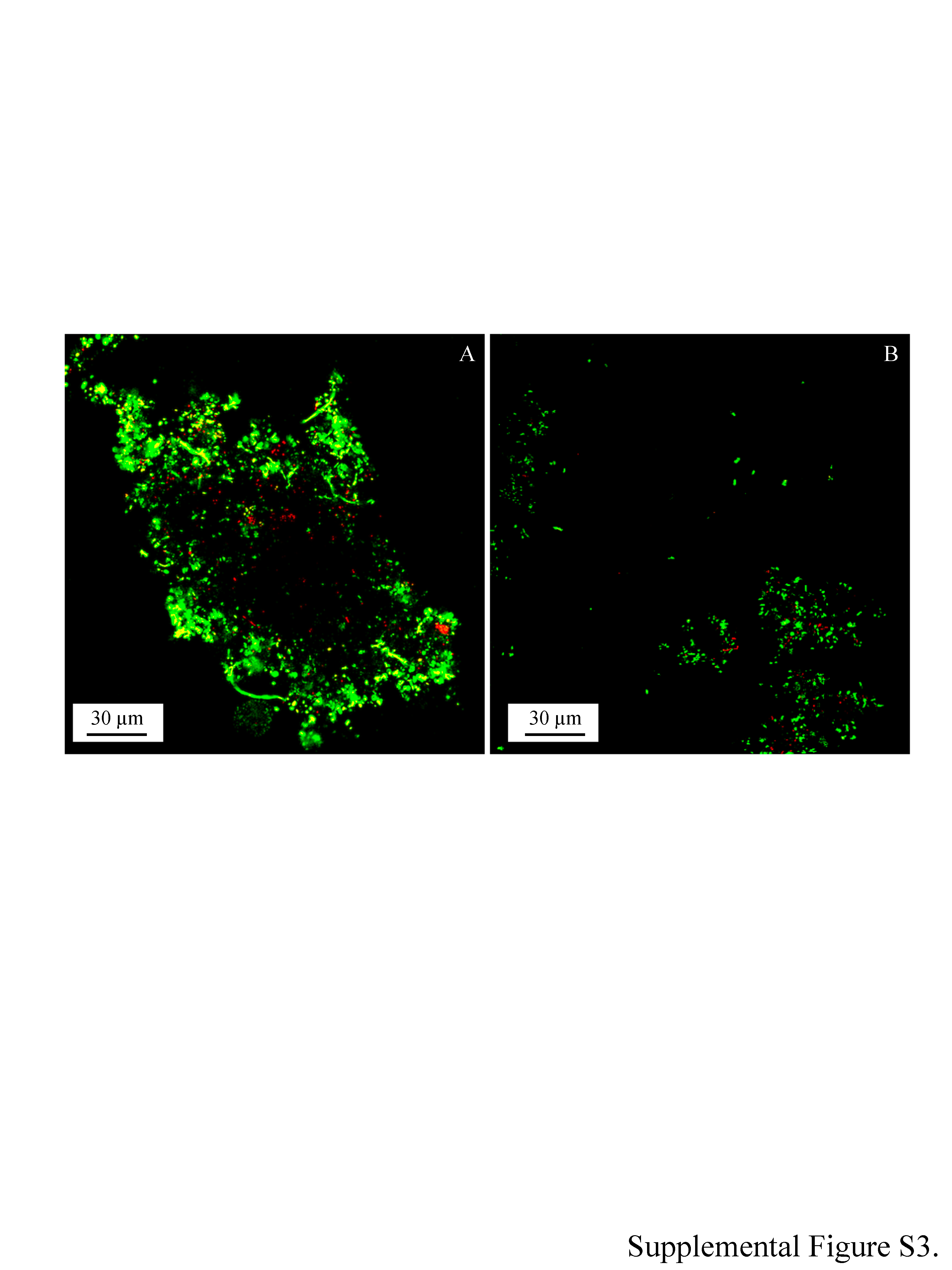

Supplement: Supplementary file 4 — Supplementary Figure S3 [file 41522_2016_10_MOESM4_ESM.tif]

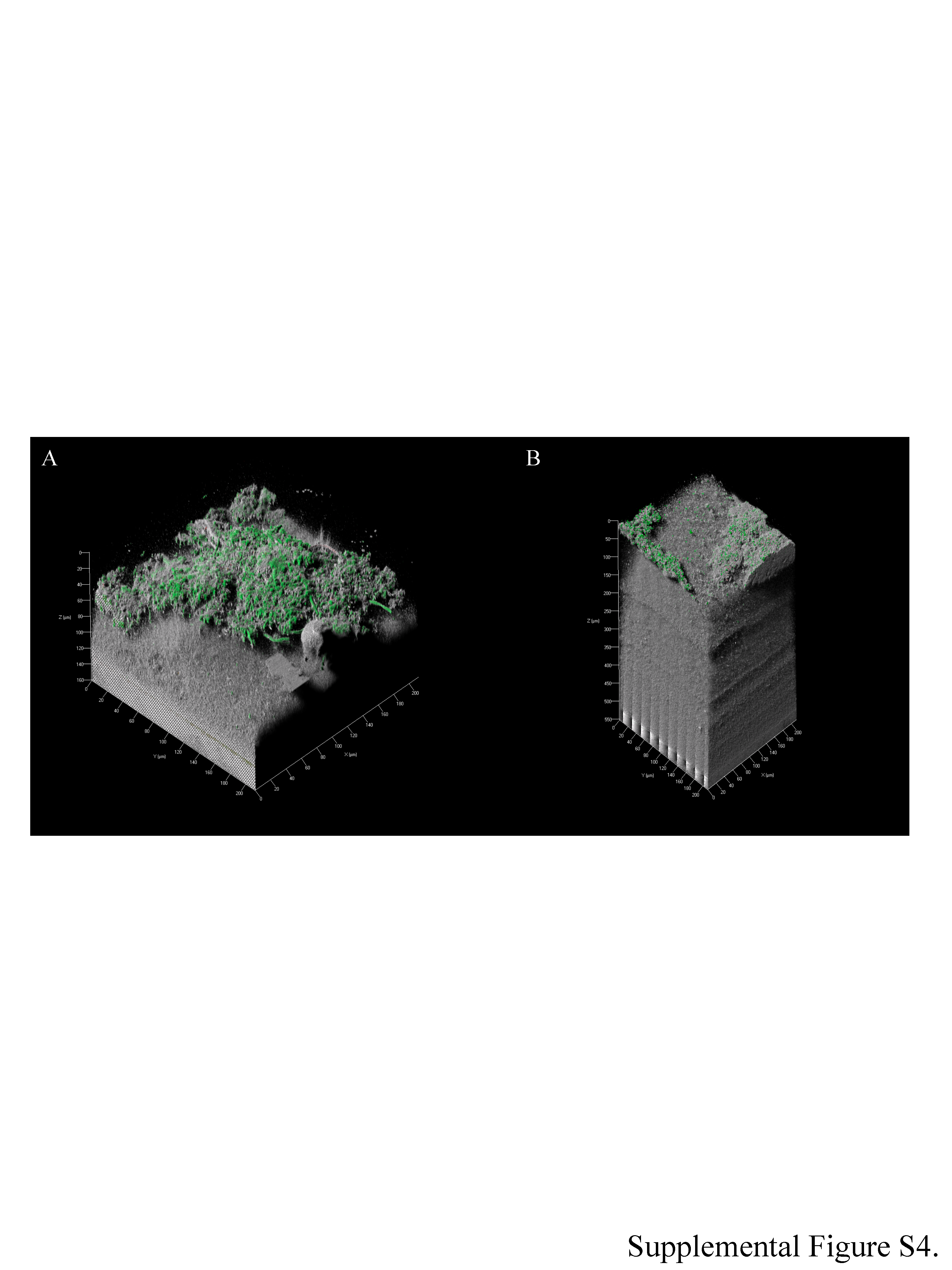

Supplement: Supplementary file 5 — Supplementary Figure S4 [file 41522_2016_10_MOESM5_ESM.tif]
